# Impact of Rural Ageing on Non-Grain Agricultural Production in China: An Analysis Based on Food Security Strategy

**DOI:** 10.3390/foods14071214

**Published:** 2025-03-30

**Authors:** Yuanzhi Guo, Yuan Tian

**Affiliations:** 1 Institute of Geographic Sciences and Natural Resources Research, Chinese Academy of Sciences, Beijing 100101, China; guoyz.16b@igsnrr.ac.cn; 2 Key Laboratory of Regional Sustainable Development Modeling, Chinese Academy of Sciences, Beijing 100101, China

**Keywords:** rural ageing, non-grain agricultural production, food security strategy, grain production, mediating effect

## Abstract

The increasing population ageing in rural China has had a far-reaching impact on agricultural production structures. However, most of the existing studies on the impact of rural ageing on NGAP are based on a micro-farmer perspective and lack consideration under macro policies. This study analyses the impact of rural ageing on NGAP within the framework of food security strategy and examines this relationship using provincial panel data based on revealing the spatial-temporal characteristics of the two. The results show that the ageing level in rural China from 2005 to 2021 exhibited a rapidly rising trend and an unbalanced distribution pattern with decreasing spatial agglomeration, while the evolution of NGAP followed an upward and then downward trend, with an increasing degree of spatial agglomeration. Under the food security strategy, rural ageing has a significant dampening effect on NGAP, which mainly stems from the partial mediating roles of the increase in the area of farmland per labourer and agricultural-related fiscal investment. In light of the future trend of population ageing in rural China, targeted measures are needed to stabilize grain production and guarantee national food security.

## 1. Introduction

Food is the top priority for human survival and development. Advances in agricultural science and technology since World War II have contributed significantly to the increase in global food production capacity [1,2,3]. However, many countries and regions still face food crises due to issues such as the spatial heterogeneity of food production conditions and unequal food distribution [4]. Low-income countries, in particular, are experiencing an alarming increase in hunger and food insecurity [5]. Meanwhile, this trend has been exacerbated by issues such as military conflicts, economic shocks, climate extremes and rising agricultural input prices, making the achievement of SDG 2 (Zero Hunger) and its related goals in 2030 a daunting challenge [5,6].

China is a populous developing country, and the Chinese government attaches great importance to food security and has made it a priority for national economic and social development [7]. Driven by a series of policies to support and benefit agriculture, China’s food production capacity has been effectively boosted [8,9], feeding 18% of the world’s population with 7% of the world’s farmland and 6% of its fresh water, thereby making a great contribution to global food security [10,11,12]. According to the national situation, grain is placed at the centre of China’s food security [12,13]. In 2022, China’s grain production increased from 113.2 million tons in 1949 to 686.53 million tons, and the per capita possession grew from 209 kg/person to 486 kg/person, above the global average [14].

Agriculture is a labour-intensive industry that requires a large amount of labour input at every stage [15,16]. However, the declining fertility rate, extended life expectancy, and massive rural exodus stemming from the rapid industrialisation and urbanisation of the country have caused rural ageing in China to become increasingly prominent, and is characterised by its large scale, high degree, and rapid pace [17,18]. A direct consequence of rural ageing is a decline in both the quantity and quality of the agricultural workforce [19]. In this context, much farmland has been abandoned or underutilised, and traditional labour-intensive agricultural production has been forced to shift to technology-intensive practices [20,21,22]. In the absence of external intervention, these changes will inevitably drive a gradual shift in agricultural production towards non-food crops to cope with labour shortages and optimize land use [21].

It has become a social consensus that rural ageing has a significant impact on the restructuring of agricultural production [23]. While there are few studies directly exploring the relationship between rural ageing and non-grain agricultural production (NGAP), research on the impact of rural ageing on grain production provides some perspective to understand this issue. For example, Qiu and Peng [24] find that rural ageing increases farmers’ possibilities to exit agricultural production through farmland abandonment and transfer, and further analysis shows that rural ageing reduces the sown areas of grain crops and their share in the total crop sown areas. Wang et al. [25] show that the higher the degree of rural ageing, the higher the technical efficiency of grain production. Han [26] states that rural ageing has an inverted U-shaped effect on grain production. When the share of rural elderly does not exceed 15.9%, rural ageing has no negative impact on grain production, while it has a significant negative impact after crossing this threshold. In general, research on the impact of rural ageing on grain production is inconclusive, with both inhibitory [24,27] and facilitative views [25,28,29], and some suggest that this impact is nonlinear [26,30]. Most of these studies are conducted at the micro-farmer level and analysed based on the economic agent hypothesis.

In long-term practice, the Chinese government has established a rural work mechanism that is coordinated by the central government, responsible to provincial governments, and implemented by municipal or county governments [31]. The food security strategy formulated by the central government is also transmitted to local governments through this mechanism. Under the system of promotion tournaments, local officials are sufficiently motivated to ensure grain production in their region as part of their political performance [32]. Therefore, it is biased to explore the impact of rural ageing on NGAP only at the micro-farmer level, and the national macro-strategy of food security should also be included in the analysis in the attempt to develop a scientific understanding of the issue. This study first explores the mechanism of rural ageing’s effect on NGAP under China’s food security strategy, then reveals the spatial-temporal evolution of rural ageing and NGAP and empirically examines the role of rural ageing on NGAP using provincial panel data. These findings can provide guidance for China to develop targeted measures to ensure food security in the context of rural ageing, and can also serve as a reference for other countries facing similar situations.

## 2. Analytical Framework

### 2.1. Food Security Strategy in China

In the early years of the People’s Republic of China, China’s food security strategy focused on ensuring grain self-sufficiency and meeting people’s basic needs through direct state intervention. After the Chinese economic reform in 1978, this strategy gradually transformed into a market-oriented one, aiming to ensure national food security through combining the development of domestic agriculture and the use of international markets. After entering the 21st century, more attention has been paid to the sustainability of grain production and the stability of the grain market, while placing greater emphasis on the impact of the international situation on the food supply. After the 18th National Congress of the CPC, the Chinese government established a food security strategy of “self-reliance, domestic dominance, ensuring productivity, moderate imports, and S&T support” based on the new concept of food security, which emphasises “ensuring basic self-sufficiency in cereals and absolute security of rations”. Thanks to decades of practice, China has explored a path to food security with Chinese characteristics.

**Figure 1:**
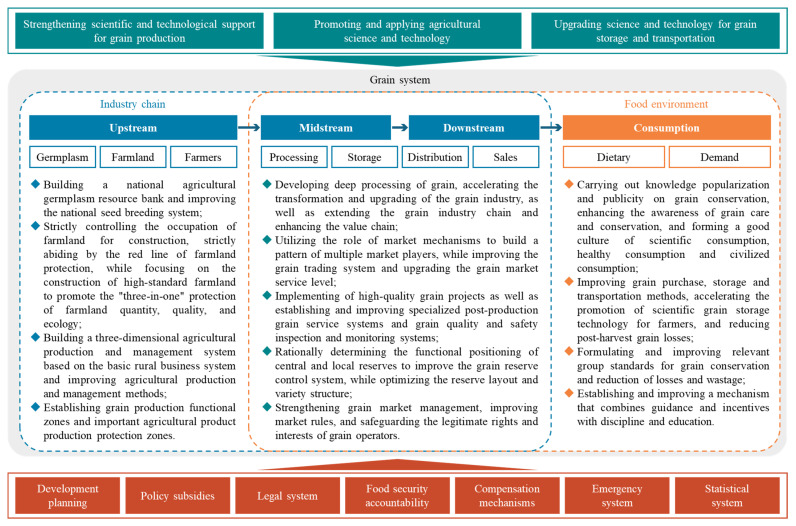
Food security system in China.

According to the basic national conditions of the primary stage of socialism and China’s huge population, ensuring grain production is the core of China’s food security strategy. Therefore, the Chinese government has identified key measures to ensure national food security from the perspective of the grain industry chain (Figure 1). For example, in the upstream, it is necessary to steadily improve grain production capacity via measures such as the construction of high-standard farmland, the development of germplasm resources, and the establishment of functional zones for grain production and protected zones for important agricultural products. In the midstream, there is a need to reasonably determine the functional positioning of central and local grain reserves, improve the reserve regulation and management mechanism, and optimize the variety structure of grain reserves. In the downstream, it is necessary to build a grain production-marketing cooperative platform and establish a synergistic promotion pattern by multiple players, thus improving the grain trading system and enhancing the service level of the grain market. In addition, the Chinese government has also identified key issues that need attention in the food environment in terms of the two dimensions of diet and demand. For instance, the government has actively launched publicity and education campaigns to raise public awareness of food conservation and established a sound mechanism combining guidance-incentives and discipline-education to reduce “waste on the table”.

To ensure the implementation of these tasks, the Chinese government has made targeted arrangements in the top-level design. Specifically, the central government has prepared a series of plans, including the Mid- and Long-term Planning Outline for National Food Security (2008–2020) and the National Plan for Agricultural Sustainable Development (2015–2030). Meanwhile, a series of laws and regulations related to food security have been implemented, in particular the Food Security Law of the PRC, which provides a legal basis for comprehensively guaranteeing national food security. A food security accountability has been established to clarify the powers and responsibilities of provincial governments in the realms of grain production, distribution, and consumption, and has been incorporated into provincial government performance appraisal system. Fiscal expenditures have also been tilted towards the issues concerning agriculture, countryside, and farmers, gradually developing a diversified policy support system focusing on agricultural inputs, price support, financial subsidies, and financial insurance. In addition, the Chinese government has established a benefit compensation mechanism between the major grain-producing and grain-marketing regions, while actively promoting the construction of an emergency response system based on improving the food statistics system.

### 2.2. Analytical Framework for the Impact of Rural Ageing on NGAP

Grain production is the process by which people utilize natural resources to obtain grain, and its development depends on the match between agricultural producers and farmland [23,33]. Compared to the stability of farmland, human initiative determines that people are the most active and decisive factor in grain production [34]. Population ageing means a decline in both the quantity and quality of the labour force, making traditional agricultural production, management and organisation methods unsustainable and leading to phenomena such as farmland abandonment [21]. Under the impetus of rapid industrialisation and urbanisation, the ageing of rural population due to the large-scale exodus of young and middle-aged labourers has led to an increasing proportion of the elderly among agricultural workers, and “elderly agriculture” has become a common phenomenon in rural China [35]. As cash crops are more demanding in terms of labour, food crops are often the priority option for “elderly agriculture”. Meanwhile, ageing farmers tend to cultivate well-located farmland, while poorly located farmland, such as those with poor irrigation and far from the home of their workers, will be abandoned [36].

To meet the strategic need to ensure national food security, the Chinese government has continuously adapted its policy system to the demographic transition occurring in rural areas. From the initial direct grain subsidies, comprehensive agricultural input subsidies, and fine seed subsidies to the current farmland fertility subsidies, social service subsidies, and agricultural machinery purchase subsidies, etc., the continuously improved agricultural subsidy policy system effectively guarantees the income of grain farmers. Public service agencies have also been established to provide farmers with services throughout the entire process of grain production, effectively substituting for labour and socializing agricultural production. Land transfer can reduce farmland abandonment, but the imperfect land transfer market in rural areas restricts the promotion of this practice [37]. Therefore, the Chinese government actively promotes the reform of the “separation of the three rights (ownership, contracting rights, and management rights) of rural land, which has stimulated farmers’ enthusiasm for transferring their management rights. The orderly transfer of land management rights has optimised land resource allocation, promoted moderate-scale agricultural operations, and realised economies of scale in grain production.

**Figure 2:**
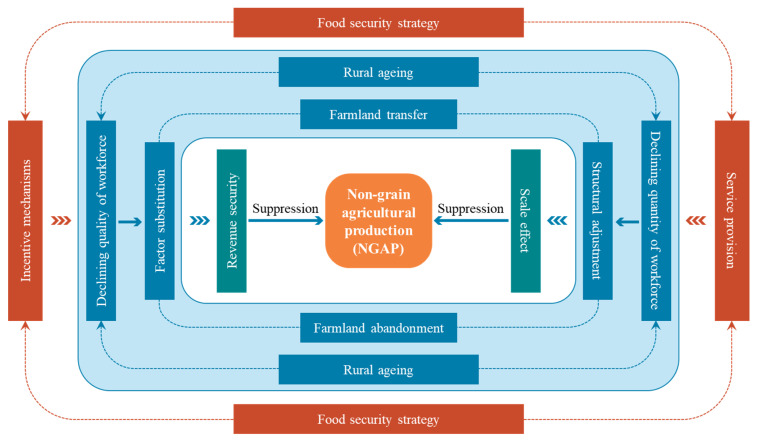
Analytical framework for the impact of rural ageing on NGAP under the food security strategy.

In general, the decline in both quantity and quality of the agricultural production labour force due to rural ageing will inevitably lead to such phenomena as farmland abandonment, land transfer, factor substitution and production restructuring. Under the national strategy of food security, a series of policies to support and benefit agriculture have increased the benefits from grain production through incentive mechanisms while ensuring the scale effect of grain production has been realised through service supply, thus discouraging NGAP (Figure 2).

## 3. Materials and Methods

### 3.1. Model and Variables

#### 3.1.1. Benchmark Model

Panel data models can control unobservable effects and mitigate the covariance problem by expanding the sample size and increasing the degrees of freedom, resulting in more accurate model estimates [38]. Here, panel data from 31 provincial administrative units in mainland China from 2005 to 2021 are employed to analyse the impact of rural ageing on NGAP, and the benchmark model is set as follows:

(1) $$NGAP_{it}=\alpha_{0}+\alpha_{1}ageing_{it}+controls+\lambda_{t}+\mu_{i}+\varepsilon_{it}$$

where *NGAP_it_* denotes the NGAP of spatial unit *i* at time *t*, which is featured by the ratio of the sown area of non-grain crops to the total crop sown area; ${ageing}_{it}$ is the rural ageing level of spatial unit *i* at time *t*, and the ratio of rural population aged 65 and over is selected as its indicator, with the old-age dependency ratio (depend) being used as a proxy variable in the robustness test; *controls* demote the control variables; $\lambda_{t}$ and $\theta_{t}$ are the temporal and spatial effects, respectively, and $\mu_{it}$ is the random perturbation term.

Agricultural production is a process that unifies natural and economic reproduction [39]. To scientifically examine the impact of rural ageing on NGAP, it is necessary to control for the effects of variables other than rural ageing. According to existing research [27,40,41,42,43] and the data available, the following ten control variables (*controls*) are included in the model analysis: ① Urbanisation rate (urban), i.e., the share of urban population to the total population in a region. ② Economic development level (econ), measured by per capita GDP, is an important indicator of regional development, along with the urbanisation rate. ③ The income of rural residents (income), measured by per capita disposable income of rural residents, is closely related to the consumption level of rural residents. ④ Educational attainment (edu), as reflected by the ratio of the illiterate to literate population aged 15 and over in rural areas, largely determines people’s ability to understand and transform the world. ⑤ Traffic conditions (traffic), characterised by road density, reveal the level of mobility factor in a region. ⑥ Labor cost (labour), represented by the average daily wage of labour, refers to the wage of a single worker for one standard working day of agricultural production and is used to reflect the opportunity cost of household labour use. ⑦ Level of agricultural mechanisation (mecha), featured by the total power of agricultural machinery per unit of sown area, is closely related to agricultural production efficiency. Unless otherwise specified, the population-related variables in this study are based on the statistical calibre of the resident population. ⑧ Farmland fragmentation (frag), defined as the fragmentation index of farmland landscape, is an important indicator reflecting agricultural production conditions. ⑨ Temperature (temp), expressed in terms of average annual temperatures. ⑩ Rainfall (rain), expressed as average annual precipitation, together with temperature, reveals meteorological conditions for agricultural production.

#### 3.1.2. Mediation Model

In the context of the government’s high priority placed on food security, farmers’ agricultural production decisions will be adjusted in response to a series of policies and measures introduced by the government, thus suppressing NGAP. Based on the above theoretical analysis and drawing on existing studies [44,45], this study further constructs a mediation model to examine the mechanism of rural ageing on NGAP:

(2) $$NGAP_{it}=\alpha_{0}+\alpha_{1}ageing_{it}+\alpha_{2}control+\varepsilon_{it}$$

(3) $$mediate_{it}=\beta_{0}+\beta_{1}ageing_{it}+\beta_{2}control+\varepsilon_{it}$$

(4) $$NGAP_{it}=\chi_{0}+\chi_{1}ageing_{it}+\chi_{2}mediate_{it}+\chi_{3}control+\varepsilon_{it}$$

where ${mediate}_{it}$ is the mediating variable, $\alpha_{1}$ and $\chi_{1}$ denote the total and direct effects of rural ageing on NGAP, respectively, and ${\lambda \beta }_{1}\times \chi_{2}$ represents the mediating effect of rural ageing on NGAP through the mediating variables.

The preceding theoretical analysis pointed out that, in the context of rural ageing, the food security strategy mainly aims to ensure that people can obtain sufficient benefits from growing grain via incentive mechanisms and service provisions. Specifically, the incentive mechanisms mainly refer to the various rewards and subsidies provided by the government to grain-growing farmers, while service provisions refer to the government’s efforts to realize the scale effect of grain production through measures such as land system reform. The implementation of these policies and measures requires substantial financial investment by the government. Therefore, the proportion of agriculture-related expenditures in local general fiscal expenditures (fiscal) and the farmland area per agricultural employee (farm) are selected as mediating variables to explore the mechanism by which rural ageing affects NGAP. In the subsequent analysis, the “three-step method” is used to examine the impact of rural ageing on NGAP and its mediating effects [45].

### 3.2. Data Sources and Profiles

“Agriculture” in this study refers to farming, that is, the cultivation of cereals, beans, tubers, cotton, oil crops, sugar crops, and other crops, the first three of which are identified as grain crops in China. According to NBS [14], the sown area of crops is an important indicator reflecting the scale of crop production and refers to the area actually sown or transplanted with crops at the end of a given production season. Any area actually planted with crops, whether on farmland or non-farmland, and regardless of size, shall be counted as sown area. All crops harvested within the current year, whether sown in the current year or harvested in the previous year, are counted as sown area, excluding crops sown in the current year but harvested in the following year.

Demographic data such as age and educational attainment were obtained from the China Population Statistics Yearbook and the China Population and Employment Statis-tics Yearbook. Of these, population census data were used for 2010 and 2020, 1% population sample survey data for 2005 and 2015, and 1‰ population change sample survey data for the remaining years. Socio-economic data, such as sown area, urbanisation rate, per capita GDP, per capita disposable income of rural residents, road density, total power of agricultural machinery, and general fiscal expenditure were collected from the China Statistical Yearbook, the China Rural Statistical Yearbook, and provincial statistical yearbooks. The average daily wage of the labour force was derived from the 2006–2022 National Compendium of Cost-Benefit Information on Agricultural Products. Data on farmland area were provided by the Sharing and Application Service Platform for Land Survey Results (https://gtdc.mnr.gov.cn/Share#/, accessed on 1 November 2024) and the China Land Resources Yearbook. Meteorological data, such as for rainfall and temperature, were sourced from the China Meteorological Data Service Centre (https://data.cma.cn/, accessed on 1 November 2024). The fragmentation index of farmland landscape was derived from Liu et al. [46]. Missing data were obtained by trend extrapolation. As a result, a total of 527 samples were obtained, and the definitions and descriptive statistics of each variable are shown in Table 1.

## 4. Results

### 4.1. Spatial-Temporal Characteristics

**Figure 3:**
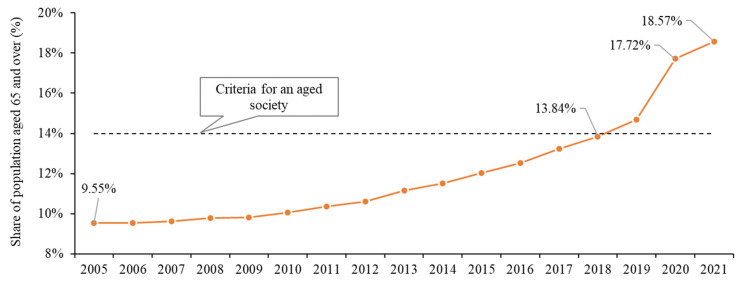
Evolution of ageing level in rural China from 2005 to 2021.

**Figure 4:**
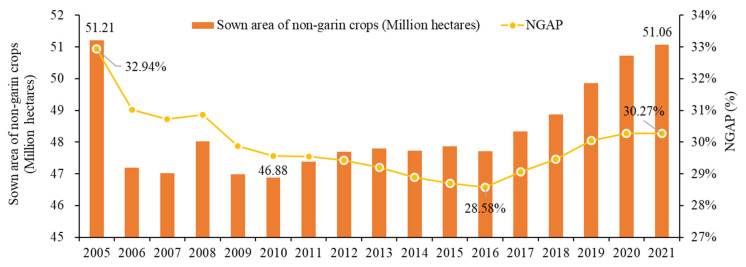
Evolution of NGAP in China from 2005 to 2021.

In 1998, the share of population aged 65 and over exceeded 7% for the first time in rural China [21], which means that rural China was formally defined as an ageing society. Since then, China’s rural population has continued to age. In 2019, this figure exceeded 14%, marking the entry of rural China into an aged society (Figure 3). By 2021, the share of China’s rural population aged 65 and over rose from 9.55% in 2005 to 18.57%, with an average annual growth rate of 4.25%. Compared with the continuous rise in rural ageing level, the evolution of China’s NGAP is generally featured by a preliminary decline and then an increase, from 32.94% in 2005 to 28.58% in 2016 and then to 30.27% in 2021. In terms of the sown area, the sown area of non-grain crops decreased rapidly in 2005–2006, then fluctuated between 47–48 million hectares in the following decade, before steadily increasing in 2016 (Figure 4).

Further, the spatial correlation of provincial rural ageing and NGAP was explored using exploratory spatial data analysis, and their spatial distribution was visualised. The results show that the Moran’s *I* index of the ratio of population aged 65 and over in rural China during the study period is significantly positive, indicating that the spatial distribution of rural ageing is featured by a “club”. Meanwhile, the Moran’s *I* index shows a general decreasing trend, suggesting that the spatial clustering of the provincial rural ageing level is gradually weakening. The Moran’s *I* index of provincial NGAP is also significantly positive, indicating a positive spatial autocorrelation. Its values are generally on the rise, indicating that the spatial clustering of provincial NGAP is increasing (Table 2).

**Figure 5:**
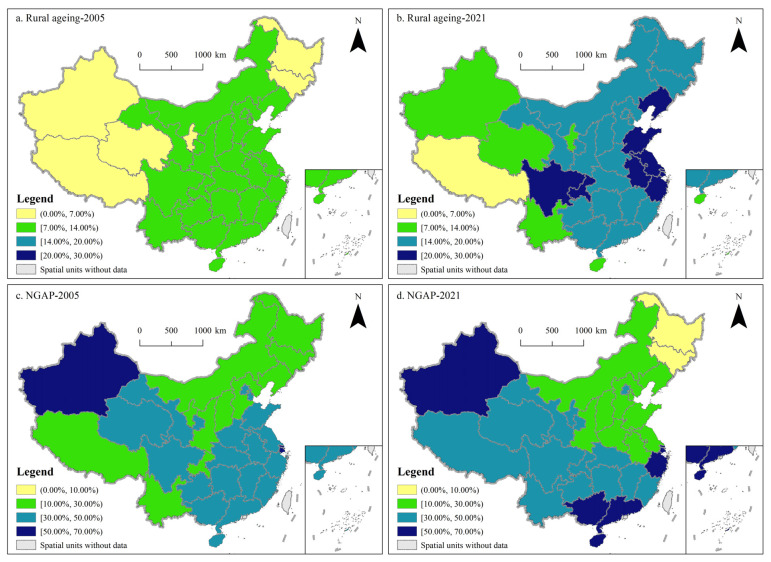
Spatial pattern of China’s rural ageing and NGAP in 2005 and 2021.

In general, a region with 7% of its population aged 65 and above is considered to be an ageing society, 14% is considered to be an aged society, and 20% is considered to be a super-aged society [47]. In 2005, the spatial gradient of rural ageing varied little across the country, with rural areas of six provinces—Ningxia, Xinjiang, Qinghai, Tibet, Heilongjiang, and Jilin—not being defined as an ageing society (Figure 5a). In 2021, rural ageing levels showed a downward trend from eastern to western China. Tibet was the only province with less than 7% of its rural population aged 65 and over, and rural areas in 5, 17, and 18 provinces were defined as ageing societies, aged societies, and super-aged societies, respectively (Figure 5b).

In terms of NGAP, its spatial distribution in 2005 showed a pattern of a northeast–southwest low-value axis crossed by a southeast–northwest high-value axis. There were 11 provinces with NGAP below 30%, of which Heilongjiang, Jilin, and Liaoning were the lowest, at 14.21%, 13.31%, and 19.61% respectively; the only provinces with NGAP above 50% were Shanghai and Xinjiang, at 58.85% and 59.99%, respectively (Figure 5c). In 2021, if a line was drawn from the Gansu–Inner Mongolia border to the Anhui–Jiangxi border, the northeast side of the line was mostly low-value provinces, while the southwest side was a cluster of high-value provinces. Specifically, Heilongjiang and Jilin saw their NGAPs fall to 3.41% and 7.53%, respectively, and they were the only two provinces with NGAP below 10%; the number of provinces with NGAP above 50% increased to six, namely Zhejiang, Guangdong, Guangxi, Shanghai, Hainan, and Xinjiang, with values of 50.03%, 50.80%, 54.30%, 55.58%, 60.37%, and 62.87%, respectively (Figure 5d).

### 4.2. Empirical Testing

To avoid the impacts of extreme values, logarithms are taken for the following five variables: econ, income, labor, mecha, and temp. Then, a test for multicollinearity was first performed before conducting the econometric analysis. The results show that lnecon has the largest variance inflation factor (VIF) of 7.615, and the rest of the variables have a VIF of less than 5. According to the empirical rule, it can be assumed that there is no multicollinearity between the variables and they are suitable for constructing the econometric model.

#### 4.2.1. Benchmark Regression

The results of the *F* test and Lagrangian Multiplier (LM) test indicate that the model analysis needs to consider individual and random effects. Meanwhile, the results of the Hausman test show that the fixed effects model is superior to the random effects model. Further, a two-way fixed effects model is employed for the final model estimation according to the R-squared values. To facilitate parameter comparisons, Table 3 presents the estimation results of the ordinary least squares (OLS) method, random effects (RE) model, fixed effects (FE1), and two-way fixed effects (FE2) model. It is found that the coefficient of rural ageing on NGAP changes from insignificant to significant with the inclusion of control variables. In terms of the impact coefficient, its value is −0.4970, which means that for every 1 percentage point increase in the proportion of rural population aged 65 and above, the NGAP level will decrease by 0.4970 percentage points. This verifies the previous theoretical analysis suggesting the negative relationship between rural ageing and NGAP.

#### 4.2.2. Robustness Test

To verify the reliability of the model’s estimation results, the robustness tests are performed in two ways. One is to replace the core explanatory variables, i.e., to use the old-age dependency ratio (depend) as a proxy variable for rural ageing. The other is to use rural ageing lagged by one period (lagged ageing) as the core explanatory variable for the current period, thus avoiding possible contemporaneous correlation between the current and residual terms. The results show that although the degree of influence of depend and lagged ageing on provincial NGAP is not the same, the direction of their effect is consistent with the results of the benchmark regression and both of them pass the significance test (Table 4), indicating that the model estimation results are robust and reliable.

#### 4.2.3. Heterogeneity Analysis

Differences in natural and human conditions determine the regional imbalances of NGAP, and there are regional differences in the impact of rural ageing on provincial NGAP due to the spatial heterogeneity of rural ageing. In accordance with the central government’s delineation of the main grain-producing areas (MGPAs), the production-marketing balance areas (PMBAs), and the main grain-marketing areas (MGMAs), this study examined the spatial heterogeneity of the impact of rural ageing on NGAP. The regression results of the subsample show that the effect of rural ageing on provincial NGAP is negative in MGPAs, PMBAs, and MGMAs, but passes the significance test only in MGPAs (Table 5). MGPAs are the core areas of China’s grain production and bear the heavy responsibility of guaranteeing national food security. Due to the severe food security situation, the Chinese government has taken a series of measures to guarantee the sown area of grain crops in MGPAs, thus inhibiting NGAP in the context of rural ageing.

Considering the differences in economic development levels of the different economic zones and their positions and tasks in terms of national development, they may also have a different impact on the externalities of rural ageing. Therefore, this study further explores the differences in the impact of rural ageing on NGAP in eastern, central, and western China. Models (4)–(6) in Table 5 show that rural ageing has a significant dampening effect on NGAP in eastern, central, and western China. This is mainly due to the superior conditions of agricultural production in eastern and central China, and the withdrawal of the elderly from agricultural production as a result of declining labour capacity is conducive to the scale effect of grain production under the strategy of food security. In the western region, elderly agriculture is prevalent, and the low labour demand of grain crops makes it the main type of elderly agriculture.

#### 4.2.4. Mediating Effect Test

Here, the farmland area per agricultural employee (farm) and the ratio of agricultural expenditure in general fiscal expenditure (fiscal) are employed as mediating variables to examine the mechanisms by which rural ageing dampens NGAP under the food security strategy (Table 6). In model (1), the variable ageing does not pass the significance test, and in model (2), lnfarm also did not pass the significance test. Therefore, it is necessary to further conduct the Sobel test; the estimation of the Sobel test passes the significance test, indicating the presence of partial mediation effects. Similarly, it can be found that the variable fiscal also plays a partial mediating role. In general, under the food security strategy, the farmland area per agricultural employee and ratio of agricultural expenditure in general fiscal expenditure play a partial mediating role in the suppression of NGAP by rural ageing.

## 5. Discussion

### 5.1. Rural Ageing and Grain Production in China

The core driver of China’s rural ageing over the past few decades has been the large-scale migration of the rural working-age population to urban areas in the course of rapid industrialisation and urbanisation [48,49]. As a result, rural ageing will inevitably bring about a shortage of agricultural labour, and this situation will continue to worsen as the level of ageing increases. Data from China’s fourth and seventh population censuses indicate that the ratios of rural population aged 45–54 and 55–64 increased from 8.02% and 6.55% in 1990 to 17.45% and 14.20%, respectively. It is expected that the ratio of people aged 65 and over in rural China will reach 30% by 2030, suggesting that China’s agricultural production will increasingly depend on the contributions of the elderly [21,23,50,51].

Although rural ageing has led to a decline in the quantity and quality of the working-age population in grain production, the increasing amount of the working-age population withdrawing from agricultural production has also created the conditions for changing traditional agricultural production, which is mainly characterised by dispersion and small scales [23]. In line with this trend, the Chinese government has introduced a series of policies and measures to guide the transfer of farmland management rights to achieve large-scale management and continuously establish and improve the agricultural socialisation service system to achieve an organic connection between small farmers and modern agriculture [52,53]. Accordingly, the sown area of grain crops has not experienced major fluctuations due to population ageing. In our previous field research, we even found that some local governments spent their own money to organize people to re-sow abandoned farmland, thus ensuring that the statistical sown area with grain crops would not be reduced. On the other hand, the sown area of non-grain crops has declined due to the labour shortage caused by rural ageing. This “rise and fall” has resulted in an increasing share of grain crops in the total sown area of crops. Therefore, within the framework of food security strategy, rural ageing suppresses NGAP through large-scale production and service supply, which is different from the findings that simply place grain production under the market mechanism in the context of rural ageing [24,27].

In 2004, the Chinese Government began to promote a nationwide policy of direct subsidies for farmers to grow grain, and the ever-improving grain subsidy policy system has played an important role in mobilizing farmers’ incentives to produce grain [54,55]. However, the government strictly controlled the increase in grain prices owing to the fundamental position of food security in national security [56]. In the case of rice, for example, the minimum purchase price for early indica rice, middle-late indica rice, and round-grained rice in 2021 rose from 2.04 yuan/kg, 2.14 yuan/kg, and 2.56 yuan/kg in 2011 to 2.44 yuan/kg, 2.56 yuan/kg, and 2.60 yuan/kg, respectively. Comparatively, the prices of agricultural inputs such as fertilizers and pesticides have continued to rise, causing China’s grain production enter an era of high costs [57]. Taking fertilizer and machinery operating costs (which account for the bulk of grain production costs) as examples, the National Compendium of Cost-Benefit Information on Agricultural Products shows that these two have increased from 128.27 yuan/mu and 98.53 yuan/mu in 2011 to 154.47 yuan/mu and 156.72 yuan/mu in 2021, respectively. In this context, it is difficult for the existing grain subsidy standards to make up for the cost-benefit gap in grain production, and the role of grain subsidy policies in curbing NGAP is gradually weakening [8,58,59].

### 5.2. Policy Implications for China’s Food Security

Thanks to its food security strategy, China has not only essentially achieved grain self-sufficiency, but has also significantly improved the quality of life and nutritional status of its people. With the improvement of living standards, people are no longer satisfied with a full stomach, and their food demand is increasingly diversified [60]. In this context, the core of China’s food security has been expanded from the grain system to the entire food system [61]. However, China’s huge population determines that the shift in the strategic focus of food security from single grain production to a diversified food supply must be based on a guaranteed grain supply. In line with the ageing trend and its challenges in rural China, several measures should be taken to promote the scale of farmland, mechanisation of production, and socialisation of services to ensure grain production.

The first is to promote the clarification of the property rights relationship of farmland. The clarification of property rights is the prerequisite for property rights protection, change, and transaction. Farmland usually involves multiple entities, such as collective economic organisations, farmers, and users, all with complex interests. Therefore, it is necessary to clearly define the power boundaries of each entity and clarify the scope of rights enjoyed to safeguard the legitimate rights and interests of all parties.

The second is to promote the marketisation of farmland transfer transactions. The market-oriented transfer of land management rights is an inevitable requirement to promote the optimal allocation of land resources in the context of rural ageing. It is necessary to improve and perfect the rural land property rights trading system to support farmland management rights to enter the market for trading, so that the land can realise its full value in the process of circulation, and increase the property income of farmers and village collectives.

The third is to establish a sound socialised service system for agricultural production. Rural ageing means a shortage of a rural working-age population. It is urgent to accelerate the cultivation and development of new-type agricultural entities. According to the needs of different entities, efforts should also be made to establish a service system that combines public and business services, coordinates special and comprehensive services, and covers the entire process of agricultural pre-production, production, and post-production.

The fourth is to optimize the grain subsidy policy system. It is necessary to adhere to the goal orientation, adjust and optimize the subsidy methods, and improve the mechanisms for protecting the interests of grain producers. In particular, the principles of market pricing and price-subsidy separation should be adhered to in order to improve the “trinity” policy system of price, subsidy, and insurance, thereby promoting the implementation of grain subsidy policies through mechanism innovation and policy integration.

## 6. Conclusions

Since the beginning of the 21st century, the phenomenon of rural ageing in China has become increasingly prominent, profoundly affecting agricultural production structures and posing a major challenge to national food security. From 2005 to 2021, the proportion of people aged 65 and over in rural China increased rapidly from 9.55% to 18.57%, while the evolution of NGAP during this period showed a downward and then an upward trend. Spatially, only rural Tibet had a population share of 65 and above below 7% in 2021, and rural areas in 18 provinces were defined as super-aged societies; the provinces with high NGAP values are concentrated in the southeast and northwest sides of the country, while those with low values were mainly located in the northeast and southwest sides. Under the food security strategy, rural ageing has a significant inhibitory effect on provincial NGAP, but this effect passed the significance test only in MGPAs, while it did not pass the significance test in PMBAs and MGMAs. The results of the mediation model show that the inhibitory effect of rural ageing on NGAP in the context of the food security strategy mainly stems from the increase in the area of farmland per labourer and agricultural-related fiscal investment, both of which play a partial mediating role. Currently, the concept of food security has been expanding, but China’s huge population size determines the importance of grain production in national food security terms. Therefore, it is necessary to take further measures to safeguard grain production, including promoting the clarification of the property rights relationship of farmland and the marketisation of farmland transfer transactions, establishing a sound socialised service system for agricultural production and optimizing the grain subsidy policy system.

This study uses provincial administrative districts as spatial units to explore the impact of rural ageing on NGAP, making it difficult to analyse the more fine-grained relationship and characteristics of the two. Therefore, some finer scale studies are worthwhile as data availability increases. Meanwhile, this study only focuses on the impact of the ageing level on NGAP, and ignores differences within the elderly group. The fact that there are significant differences in both labour capacity and technology adoption among the low-age, middle-age, and high-age elderly determines the need for further analysis of the scientific issues of this study from the perspective of intra-group differences. Finally, this study provides some policy insights on the research findings, but they are mostly at the macro level and need to be further refined in light of local conditions when they are implemented.

## Figures and Tables

**Table 1:** Summary statistics of the variables.

Variables	Definition	Min	Max	Mean	SD
NGAP	Ratio of the sown area of non-grain crops to the total crop sown area (%)	2.92%	67.18%	34.76%	0.133
ageing	Ratio of population aged 65 and over to total population (%)	5.02%	26.80%	11.41%	0.040
depend	Ratio of population aged 65 and over to population aged 15–64 (%)	7.04%	45.80%	16.58%	0.066
urban	Ratio of urban population to regional total population (%)	20.79%	89.60%	54.86%	0.147
econ	Per capita GDP (Yuan/person)	5052	183,980	45,415	28,990
income	Per capita disposable income of rural residents (Yuan/person)	1877	112,950	10,387	7797
edu	Illiterate population aged 15 and over as a share of total population (%)	2.03%	47.90%	9.99%	0.075
traffic	Length of highway per unit area of a region (km/km^2^)	0.036	2.233	0.855	0.503
labor	Average daily wage of labour (Yuan/day)	9.8	162.9	54.7	33.0
mecha	Total power of agricultural machinery per unit of sown area (kWh/ha)	2.105	24.626	6.405	3.380
frag	Fragmentation index of farmland landscape (-)	0.318	0.538	0.438	0.052
temp	Average annual temperatures (°C)	2.582	25.839	13.725	5.462
rain	Average annual precipitation (m)	0.001	0.006	0.003	0.001
fiscal	Ratio of agricultural expenditure in general fiscal expenditure (%)	1.60%	20.38%	10.61%	0.037
farm	Farmland area per agricultural employee (mu/person)	2.509	49.901	9.138	6.904

**Table 2:** The Moran’s *I* index of China’s provincial rural ageing and NGAP from 2005 to 2021.

Rural ageing	2005	2006	2007	2008	2009	2010	2011	2012	2013
	0.2451 ***	0.1988 ***	0.2169 ***	0.2060 ***	0.2136 ***	0.1769 ***	0.1312 **	0.0885	0.1278 **
	2014	2015	2016	2017	2018	2019	2020	2021	
	0.1248 **	0.1507 **	0.1146 *	0.1436 **	0.1135 *	0.1145 *	0.1477 **	0.1559 **	
NGAP	2005	2006	2007	2008	2009	2010	2011	2012	2013
	0.1114 *	0.1389 **	0.1757 ***	0.1784 ***	0.2117 ***	0.2105 ***	0.2037 ***	0.2120 ***	0.2087 ***
	2014	2015	2016	2017	2018	2019	2020	2021	
	0.1913 ***	0.2008 ***	0.1901 ***	0.1979 ***	0.2067 ***	0.2078 ***	0.1886 ***	0.2128 ***	

Note: * *p* < 0.1, ** *p* < 0.05, *** *p* < 0.01.

**Table 3:** Benchmark regression results.

Variables	Model (1) OLS	Model (2) OLS	Model (3) RE	Model (4) FE1	Model (5) FE2
ageing	−0.1448	−1.0258 ***	−0.3175 ***	−0.3210 ***	−0.4970 ***
	(−1.0046)	(−5.9452)	(−3.5818)	(−3.6123)	(−3.7529)
urban		0.0766	0.0697	0.0722	−0.1403
		(1.0992)	(0.9386)	(0.9294)	(−1.2985)
lnecon		0.1375 ***	0.0838 ***	0.0752 ***	0.1207 ***
		(5.1854)	(5.1068)	(4.5951)	(6.4053)
lnincome		−0.0768 **	−0.0143	−0.0087	−0.0105
		(−2.4401)	(−1.0992)	(−0.6755)	(−1.4250)
edu		0.4793 ***	0.0503	0.0496	−0.2111 **
		(5.6762)	(0.6818)	(0.6649)	(−2.1093)
traffic		−0.0472 **	−0.0436 ***	−0.0330 **	−0.0111
		(−2.5136)	(−3.1704)	(−2.3243)	(−0.5572)
lnlabor		0.0196	−0.0408 ***	−0.0377 ***	−0.0620 ***
		(0.9267)	(−3.5649)	(−3.2901)	(−4.1998)
frag		0.3346 **	−0.5102 ***	−0.9289 ***	−0.9503 ***
		(2.4944)	(−2.6281)	(−4.2294)	(−3.7463)
lnmecha		−0.0419 ***	0.0472 ***	0.0485 ***	0.0513 ***
		(−3.2382)	(4.1825)	(4.2703)	(3.9099)
rain		7.1784	3.7927	1.5349	−5.3239
		(1.4798)	(1.0753)	(0.4262)	(−1.4101)
lntemp		0.1617 ***	0.0688 ***	0.0114	−0.0168
		(9.9890)	(2.7607)	(0.3696)	(−0.5268)
Constant	0.3642 ***	−0.9125 ***	−0.2642 *	0.0851	0.2159
	(20.8886)	(−4.4937)	(−1.6518)	(0.4912)	(0.9421)
N	527	527	527	527	527
R^2^	0.0019	0.4541	0.2010	0.2129	0.9510

Note: * *p* < 0.1, ** *p* < 0.05, *** *p* < 0.01; and *t* statistics in parentheses.

**Table 4:** Robustness test results.

Variables	Model (1) Depend	Model (2) Lagged Ageing
depend	−0.1817 ***	
	(−3.5915)	
urban	0.0897	0.0321
	(1.1328)	(0.3890)
lnecon	0.0746 ***	0.0851 ***
	(4.5442)	(5.1808)
lnincome	−0.0102	−0.0057
	(−0.8027)	(−0.4603)
edu	0.0566	−0.0490
	(0.7576)	(−0.6180)
traffic	−0.0319 **	−0.0044
	(−2.2299)	(−0.2163)
lnlabor	−0.0381 ***	−0.0459 ***
	(−3.3349)	(−3.9642)
frag	−0.9176 ***	−0.9432 ***
	(−4.1886)	(−4.3581)
lnmecha	0.0491 ***	0.0454 ***
	(4.3284)	(4.0071)
rain	1.6710	1.3805
	(0.4632)	(0.3941)
lntemp	0.0065	0.0541 *
	(0.2114)	(1.7439)
lagged ageing		−0.4545 ***
		(−4.5713)
Constant	0.0959	−0.0914
	(0.5525)	(−0.5233)
N	527	496
R^2^	0.2126	0.2268

Note: * *p* < 0.1, ** *p* < 0.05, *** *p* < 0.01; and *t* statistics in parentheses.

**Table 5:** Estimation results for different regions.

Variables	Model (1) MGPAs	Model (2) PMBAs	Model (3) MGMAs	Model (4) Eastern China	Model (5) Central China	Model (6) Western China
ageing	−0.4309 ***	−0.1272	−0.1922	−0.4366 ***	−0.9331 ***	−0.2378 *
	(−3.6819)	(−0.8884)	(−0.8849)	(−2.8698)	(−5.8140)	(−1.9385)
urban	0.0377	0.4663 ***	−0.0127	−0.2705 **	0.2048	0.1554
	(0.3464)	(3.3028)	(−0.0674)	(−2.4095)	(1.6478)	(1.1904)
lnecon	0.0634 ***	0.0252	0.0758	0.0519	0.0344	0.0136
	(3.2168)	(1.2923)	(1.3873)	(1.6061)	(1.5754)	(0.7763)
lnincome	−0.0072	−0.0666 **	0.0457	0.0590	−0.0040	0.0200
	(−0.6700)	(−2.5294)	(0.8019)	(1.4278)	(−0.4233)	(0.8787)
edu	0.1998	−0.0369	0.1830	0.1313	0.0572	0.0208
	(1.6446)	(−0.5557)	(0.9659)	(0.9061)	(0.4182)	(0.3517)
traffic	−0.0364 **	0.0406 **	−0.1308 ***	−0.1138 ***	−0.0302 *	0.0904 ***
	(−2.2931)	(2.0504)	(−3.2186)	(−4.6368)	(−1.7098)	(5.1259)
lnlabor	−0.0279 **	0.0058	−0.0584	−0.0214	−0.0141	−0.0118
	(−2.3846)	(0.3814)	(−1.6543)	(−0.8596)	(−1.0959)	(−0.9051)
frag	−1.3562 ***	−0.1605	−1.1400 **	−1.8512 ***	−2.5657 ***	0.2406
	(−4.4436)	(−0.6609)	(−2.0462)	(−4.3307)	(−5.8632)	(1.1294)
lnmecha	−0.0032	0.0419 ***	0.1921 ***	0.1524 ***	−0.0013	−0.0434 **
	(−0.2335)	(3.0551)	(6.7401)	(7.0910)	(−0.1237)	(−2.5248)
rain	5.7395	−0.1455	2.0210	−0.2191	8.8094 **	2.4841
	(1.4047)	(−0.0262)	(0.2986)	(−0.0412)	(2.2509)	(0.4960)
lntemp	−0.0103	0.0266	0.0037	0.0142	0.0030	0.0081
	(−0.3692)	(0.6683)	(0.0283)	(0.1724)	(0.1118)	(0.2488)
Constant	0.3835 *	0.3978 *	−0.2627	0.1687	1.0106 ***	−0.0609
	(1.8995)	(1.7642)	(−0.4912)	(0.4818)	(3.9331)	(−0.3032)
N	221	187	119	187	136	204
R^2^	0.4538	0.5773	0.5253	0.5328	0.5923	0.6282

Note: * *p* < 0.1, ** *p* < 0.05, *** *p* < 0.01; and *t* statistics in the parentheses. MGPR includes Heilongjiang, Jilin, Liaoning, Inner Mongolia, Hebei, Henan, Shandong, Jiangsu, Anhui, Jiangxi, Hubei, Hunan, and Sichuan; PMBR includes Shanxi, Ningxia, Qinghai, Gansu, Tibet, Yunnan, Guizhou, Chongqing, Guangxi, Shaanxi, and Xinjiang; and MGMR includes Beijing, Tianjin, Shanghai, Zhejiang, Fujian, Guangdong, and Hainan. Eastern China includes Beijing, Tianjin, Hebei, Liaoning, Shanghai, Jiangsu, Zhejiang, Fujian, Shandong, Guangdong, and Hainan; Central China includes Heilongjiang, Jilin, Shanxi, Anhui, Jiangxi, Henan, Hubei, and Hunan; and Western China includes Inner Mongolia, Guangxi, Chongqing, Sichuan, Guizhou, Yunnan, Tibet, Shaanxi, Gansu, Qinghai, Ningxia, and Xinjiang.

**Table 6:** Estimation results of the mediation model.

Variables	Per Labourer Farmland Area		Share of Agricultural Expenditure	
	Model (1)	Model (2)	Model (3)	Model (4)
	lnfarm	NGAP	fiscal	NGAP
ageing	1.1294	−0.4495 **	−0.0597	−0.4929 **
	(1.6693)	(−2.0811)	(−0.8916)	(−2.1110)
urban	2.1072 ***	−0.0516	0.0744 **	−0.1454
	(2.9053)	(−0.1955)	(2.1150)	(−0.4924)
lnecon	−0.1517	0.1143 ***	−0.0302 **	0.1228 ***
	(−1.1386)	(3.2808)	(−2.6773)	(3.3271)
lnincome	−0.0196	−0.0114	0.0152 **	−0.0116
	(−0.5410)	(−0.7402)	(2.6203)	(−0.7505)
edu	−0.2184	−0.2203	−0.0674	−0.2064
	(−0.4371)	(−1.4183)	(−1.3849)	(−1.3117)
traffic	−0.0436	−0.0130	−0.0303 ***	−0.0090
	(−0.4126)	(−0.3433)	(−2.8736)	(−0.2185)
lnlabor	0.0150	−0.0613 *	0.0065	−0.0624 *
	(0.1156)	(−1.8815)	(0.7557)	(−2.0372)
frag	−1.0406	−0.9941 **	−0.4056 ***	−0.9222 *
	(−0.4700)	(−2.0700)	(−2.8771)	(−1.8273)
lnmecha	−0.1315 *	0.0458	0.0127 *	0.0504
	(−1.8304)	(1.4331)	(1.8685)	(1.5312)
rain	25.3116 **	−4.2584	0.7511	−5.3761 *
	(2.1191)	(−1.5053)	(0.5121)	(−1.7623)
lntemp	0.0247	−0.0157	−0.0031	−0.0165
	(0.2205)	(−0.5072)	(−0.2519)	(−0.5119)
lnfarm		−0.0421		
		(−1.5239)		
fiscal				0.0695
				(0.3601)
Constant	2.8564	0.1114	0.3630 **	−0.0341
	(1.6153)	(0.2786)	(2.2953)	(−0.0803)
N	527	527	527	527
R^2^	0.7105	0.3473	0.7515	0.3324

Note: * *p* < 0.1, ** *p* < 0.05, *** *p* < 0.01; and *t* statistics in the parentheses.

## Data Availability

The associated dataset of the study is available upon request to the corresponding author.
